# Disaster-Driven Evacuation and Medication Loss: a Systematic Literature Review

**DOI:** 10.1371/currents.dis.fa417630b566a0c7dfdbf945910edd96

**Published:** 2014-07-18

**Authors:** Sae Ochi, Susan Hodgson, Owen Landeg, Lidia Mayner, Virginia Murray

**Affiliations:** Department of Internal Medicine, Soma Central Hospital, Soma, Fukushima, Japan; School of Public Health, Imperial College London, London, UK; Extreme Events and Health Protection, Public Health England, London, UK; Flinders University Disaster Research Centre, Flinders University, Adelaide, South Australia, Australia; Extreme Events and Health Protection, Public Health England, London, UK

## Abstract

AIM: The aim of this systematic literature review was to identify the extent and implications of medication loss and the burden of prescription refill on medical relief teams following extreme weather events and other natural hazards. METHOD: The search strategy followed the Preferred Reporting Items for Systematic Reviews and Meta-Analyses (PRISMA). Key health journal databases (Medline, Embase, PsycINFO, Maternity and Infant Care, and Health Management Information Consortium (HMIC)) were searched via the OvidSP search engine. Search terms were identified by consulting MeSH terms. The inclusion criteria comprised articles published from January 2003 to August 2013, written in English and containing an abstract. The exclusion criteria included abstracts for conferences or dissertations, book chapters and articles written in a language other than English. A total of 70 articles which fulfilled the inclusion criteria were included in this systematic review. RESULTS: All relevant information was collated regarding medication loss, prescription loss and refills, and medical aids loss which indicated a significant burden on the medical relief teams. Data also showed the difficulty in filling prescriptions due to lack of information from the evacuees. People with chronic conditions are most at risk when their medication is not available. This systematic review also showed that medical aids such as eye glasses, hearing aids as well as dental treatment are a high necessity among evacuees. DISCUSSION: This systematic review revealed that a considerable number of patients lose their medication during evacuation, many lose essential medical aids such as insulin pens and many do not bring prescriptions with them when evacuated.. Since medication loss is partly a responsibility of evacuees, understanding the impact of medication loss may lead to raising awareness and better preparations among the patients and health care professionals. People who are not prepared could have worse outcomes and many risk dying when their medication is not available.

## Introduction

After an extreme weather event or other natural hazard, the continuity of routine care is one of many challenging aspects of post disaster healthcare. Although a disaster can impact on all available services, healthcare facilities can be overwhelmed reducing their ability to maintain normal function. Structural and non-structural damage to their buildings, creating an insecure environment for hospital staff, and disruption of supply chains, all lead to closure of wards for new admissions, or even evacuation of patients and staff, at a time when they are critical for those who are injured by the disaster. Recognising the impact of extreme events on healthcare facilities, normal functioning is still required for people needing routine health management such as oncological treatment, dialysis and maternity care, as well as those people with chronic conditions who require daily medication to maintain their wellbeing.

Long-term non-communicable diseases 1 are increasing with a growing and ageing world population and in 30 years from 1990, NCD’s are estimated to increased 1.8 times 2. Ensuring continuation of routine care for chronic conditions will be an increasing burden during and post disaster periods both in developing and developed countries 3. Interruption of routine medication lead can lead to an exacerbation of chronic conditions such as insulin-dependent diabetes 4 and infectious diseases for example tuberculosis 5. This can also potentially cause secondary life-threatening outcomes as a result of the deterioration of chronic conditions such as ischemic heart diseases among patients with hypertension 6, and low compliance to medication regimens in the future 7 ^,^ 8.

During any disaster, medication maintenance is problematic due to people not having adequate dosages for a sufficient period of time, not having prescriptions with them, not remembering the medication they are on and more likely not having any medication with them at all. These people have been described as ‘drug refugees’. In the Great East Japan Earthquake in 2011, for example, a large number of ‘drug refugees’ were reported 9, and at least 283 people were reported to have died from the exacerbation of pre-existing conditions due to lack of access to healthcare 10. The health impacts on drug refugees has had, little research conducted among the affected population.

Since medication loss is partly a responsibility of evacuees, understanding the impact of medication loss may lead to raising awareness and better preparations among the patients and health care professionals. The aim of this systematic literature review was to identify the extent and implications of medication loss and the burden of prescription refill on medical relief teams following extreme weather events and other natural hazards.

## Methods

The search strategy followed the Preferred Reporting Items for Systematic Reviews and Meta-Analyses (PRISMA) statement where applicable, and this checklist was used in designing and reporting our review 11.

**Identification**

The key health journal databases (Medline, Embase, PsycINFO, Maternity and Infant Care, and Health Management Information Consortium (HMIC)) were searched via the OvidSP search engine. Search terms were identified by consulting MeSH terms. The validity of the search was confirmed by comparing the generated results to articles obtained from expert consultation and ‘snow-ball’ search). After this pilot search, it was revealed that using only MeSH terms was not sufficient to identify all relevant articles. Therefore, key words related to disaster and medication were added to MeSH term search as shown in Table 1. The relevant articles were searched by combining [Terms for disasters] AND [Terms for medications].

**Table 1. Search terms used and limitations:** 

Search Area	Term Category	Terms
**MeSH terms**	Disaster	Disasters Disaster medicine Disaster planning Emergencies Emergency shelter Relief work
	Medication	Chronic disease Community-based participatory research “Delivery of health care” Drug prescriptions Drug utilization “Health services needs and demand” Health services accessibility Medication adherence Needs assessment “Patient acceptance of health care” Patient compliance Pharmaceutical preparations Prescriptions Prescription drugs
**Key words**	Disaster	Disaster$ Earthquake/Earthquakes Evacu$ Flood/Floods Hurricane/Hurricanes Landslide/Landslides Tsunami Typhoon/Typhoons Volcan$ Wildfire/Wildfires
	Medication	Prescri$ Chronic disease/diseases Chronic condition/conditions Medication/medications Needs assessment/assessments
**** **Limitations specified when searching the literature: ** Human; Date of publication from 2003-2013; English Language; with abstract		

**Eligibility criteria**

**1) Inclusion criteria**

Articles were eligible if they were published over the period from January 2003 to August 2013, were written in English, and included an abstract. We limited the search period to10 years because technology and needs for chronic diseases such as dialysis and home oxygen therapy treatments have changed over this period of time. Eligible articles described the following: (i) evacuees’ actions of bringing prescription medications with them; (ii) burden of prescription refills or prescription of medication for chronic conditions within relief activities after disasters, and/or (iii) disruption of medications due to evacuees not bringing their medications.

**2) Exclusion criteria**

Articles and papers were excluded if they were (i) abstracts for conferences or dissertations; (ii) chapters of books; and (iii) articles written in a language other than English.

**Study selection**

The search was conducted on 5^th^ September, 2013 and generated 5,382 results of which 1,652 were duplicates and removed, leaving 3,730 records, Out of these records 2,961 were apparently irrelevant when screened by title exclusion criteria. Hence, the initial screening by title identified 811 records as relevant and for these remaining articles, abstracts were checked independently by SO and other co-authors (SH and LM). Abstract eligibility screening excluded a further 513, however, given the wide range of sources searched, an additional 66 abstracts were added as a result of citation searching, shown as ‘secondary screening’ in Figure 1. Overall, a total of 364 articles were identified. The full-text articles eligibility screening identified those not fulfilling the inclusion criteria which totalled 294, thus leaving a total of 70 articles for this systematic review (Figure 1).

**Study selection flow chart:**
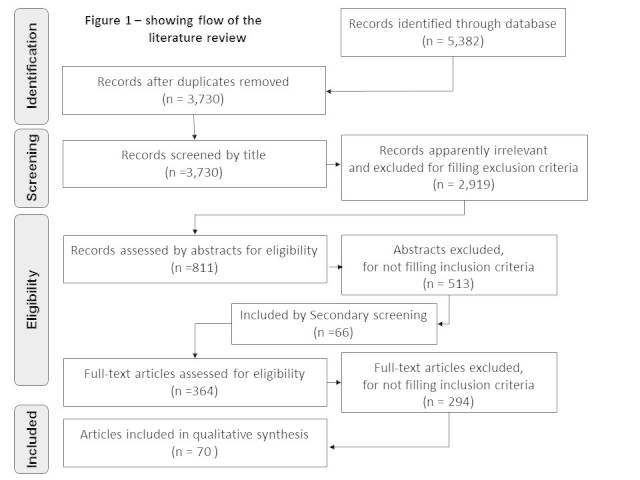

**Risk of bias and data synthesis**

Most of the data collected were from observational studies using convenience population samples because obtaining robust data from appropriate population samples is almost impossible immediately after an extreme event. Due to the heterogeneity of the data, no formal assessment of bias in each study was made. In addition, due to the wide variety of the targeted populations, statistical data synthesis was considered inappropriate.

## Results

This is the first systematic review that has addressed the topic of medication needs in disaster driven evacuation. Most of these publications did not focus on the medications needs from disaster driven evacuation thus the information was limited and often not covered comprehensively. Although the results are provided in detail below the findings are used principally to inform a detailed commentary of the assimilated results from the many publications identified.

From the MeSH terms, 70 papers were identified of which 69 articles were related to extreme events. These events showed a wide range of disasters occurring from 1992 to 2011 and included 14 hurricanes, typhoons or cyclones; eight earthquakes; two flooding; one wildfire; and one power outage and one conflict (Table 2). There were 29 (44%) articles that reported on the impacts from the 2005 Hurricane Katrina. Additionally 54 (78%) of the disasters identified occurred in the United States of America (USA), a total of nine countries were found to have reported these extreme events.

**Table 2. Chronological list of the reviewed events:** 

**Year**	**Month**	**Name of events**	**Country**	**No. of articles**
1992	August	Hurricane Andrew	US	4
1992	September	Hurricane Iniki	US	3
1994	January	Northridge earthquake	US	1
1995	January	Hanshin earthquake	Japan	1
1998-1999	-	Kosovo crisis	Kosovo	2
2001	January/February	El Salvador earthquakes	El Salvador	1
2001	June	Tropical Storm Allison	US	3
2003	August	New York Blackout	US	1
2003	December	Bam earthquake	Turkey	1
2004	April	Typhoon Sudal	Micronesia	1
2004	August	Hurricane Charley	US	1
2004	August-September	Hurricane Frances	US	1
2004	September	Hurricane Ivan	US	1
2004	September	Hurricane Jeanne	US	1
2004	October	Mid-Niigata Prefecture earthquake	Japan	1
2005	August	Hurricane Katrina	US	29
2005	September	Hurricane Rita	US	2
2005	October	Pakistan earthquake	Pakistan	1
2005	October	Hurricane Wilma	US	2
2006	October	Flash flood in Japan	Japan	1
2007	October-November	California Wildfire	US	1
2008	June	Flooding in Iowa	US	1
2008	August-September	Hurricane Gustav	US	1
2008	September	Hurricane Ike	US	1
2010	January	Haiti earthquake	Haiti	1
2011	February	Cyclone Yasi	Australia	1
2011	March	Great East Japan Earthquake	Japan	3
-	-	Not specific		2
**Total**		**27 events**	**9 countries**	**69 articles**

Table 3 is a summary of articles related to medication loss and interruption of care listed by evacuees, condition type and population based studies and lists chronologically the type of disaster, study method, sample and sample size for this systematic review where relevant. There are three sections within Table 3 and within each section there is an account of the outcome of each report relating to medication loss, medication unavailability and not having an adequate supply.

**Table 3**

**Figure:**
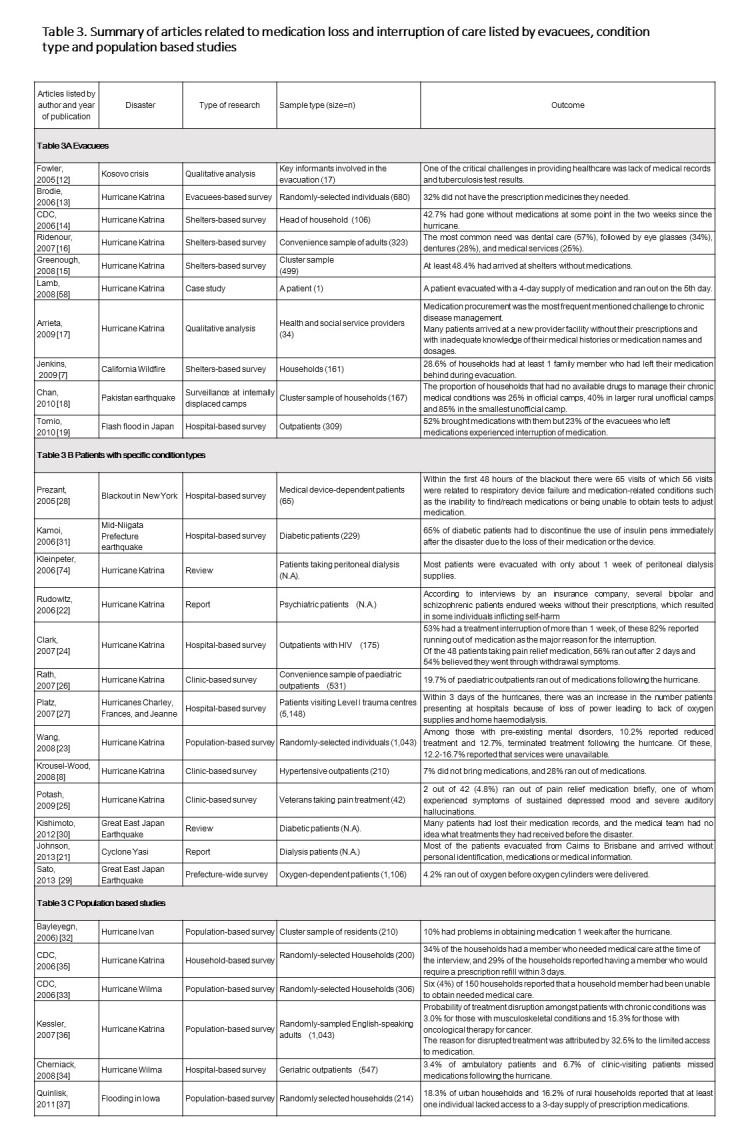

Table 3A Evacuees

A significant proportion of evacuees from, or residents at, disaster areas lost their medication.

In the first study chronologically, Fowler et al.,^12^ reported from interviews with humanitarian workers involved in the evacuation after the Kosovo crisis in 1999, which revealed that one of the critical challenges in providing healthcare was the lack of medical records and tuberculosis (TB) test results. From the six studies involving Hurricane Katrina it is possible to determine that a major proportion of people did not have adequate medication supplies, an issue identified in five of these papers. In three studies the percentage of those without medication were identified, namely 32% by Brodie et al. 13; 42.7% by CDC 14 and 48.4% by Greenough et al. 15. In addition the questionnaire-based survey amongst evacuees who arrived at a family assistance centre two weeks after Hurricane Katrina revealed that the 42.7% had run out of their medications even when they brought them 14. In another study Ridenour et al., 16 reported that, from a convenience sample of 323 adults, 57% of people required dental care, 34% eye glasses, 28% denture and 25% medical services. In the final paper in this category from Hurricane Katrina, Arrieta et al. 17 reported that medication procurement was the most frequent mentioned challenge to chronic disease management and that ‘ *many patients arrived at a new provider facility without their prescriptions and with inadequate knowledge of their medical histories or medication names and dosages’.*

The remaining publications in this section related to a Californian wildfire, the Bam earthquake and flash floods in Japan. Data from California showed that patients did not have their medications with them during evacuation for one family member in 28.6% of households 7. Information from a cluster sample of households following the Bam earthquake reported that 25% of people in in official camps, 40% in larger rural unofficial camps and 85% in the smallest unofficial camp had no available drugs to manage their chronic medical conditions 18. After the flash floods in Japan in 2006, 48% of the evacuees left their medication and 88% left their prescription records behind 19.

Table 3B Patients with specific condition types

In this section, the focus was on people with specific medical conditions and availability of continued medication treatment following an extreme event. Eight articles focussed on hurricanes in the USA, seven were related to Hurricane Katrina and one article covered the impact of three consecutive hurricanes in Florida. Other articles in this section covered a power outage in New York, earthquakes in Japan and a cyclone in Queensland, Australia.

Following Hurricane Katrina evacuees on peritoneal dialysis were asked to bring with them approximately 1 week supply of personal medical supplies 20; however, some dialysis patients evacuated from Cairns to Brisbane during cyclone Yasi arrived without personal identification, medications or medical information 21.

Mental health impact reports following Hurricane Katrina showed that some psychiatric patients inflicted self-harm as a result of not having prescriptions filled 22. A telephone interview with randomly-selected English-speaking adult Katrina survivors in New Orleans, Alabama, Louisiana, and Mississippi indicated that 21.3% had a pre-existing mental disorder, of whom 10.2% had reduced, and 12.7% terminated, their treatment because of the hurricane 23.

Again from Hurricane Katrina reports, treatment interruption occurred for more than 1 week for 53% of people with HIV, of which 82% reported the interruption being due to their medication running out, further, 48 patients from this study who were taking pain relief medication 56% ran out after 2 days and 54% believed they were going through withdrawal symptoms 24. In another study, Potash et al. 25 reported that 4.8% of people ran out of their pain relief medication which caused one person to experience severe side effects from not having this medication. Rath et al. 26 reported that 19.7% of paediatric outpatients ran out of medications, while 7% of patients with hypertension did not bring their medication with them and 28% ran out of their supply 8.

In Florida following the three Hurricanes of Charley, Frances and Jeanne there was an increase in the number patients presenting at hospitals because of loss of power leading to lack of oxygen supplies and home haemodialysis 27.

During the New York blackout, within 48 hours the Emergency Department of Montefiore Medical Center reported that 56 out of 65 visits were related to respiratory device failure and that medication-related problems were due to people unable to find or reach medications or were unable to obtain tests needed to adjust medications such as warfarin 28.

In Japan during the following the Great East Earthquake 4.2% of people ran out of oxygen before a new supply of oxygen cylinders could be delivered 29. Kishimoto and Noda 30 noted that many diabetic patients had lost their medication records, and the medical team could not determine what treatments they had received before the disaster. After the Mid-Niigata earthquake in Japan in 2004, hospital-based survey reported that 65% of patients with type 1 diabetes with insulin therapy were unable to continue the use of insulin pens due to medication loss immediately after the earthquake 31.

Table 3C Population based studies

In this section six reports focus on groups of people and their medication treatment in the USA following Hurricanes Ivan, Katrina and Wilma and the flooding in state of Iowa.

Bayleyegn et al. 32 reported that 10% of a cluster sample of 210 people had problems obtaining medication 1 week following Hurricane Ivan. After Hurricane Wilma, six (4%) of 150 households surveyed, reported that a household member had been unable to obtain needed medical care 33 and Cherniack et al. 34 reported that 10.1% of patients missed medications after the hurricane. Following Hurricane Katrina surveys showed that 34% of the households had a member who needed medical care at the time of the interview, and who would require a prescription refill within 3 days 35. Kessler et al. 36 noted that treatment was disrupted for patients with musculoskeletal conditions (3.0%) and those with oncological conditions (15.3%) and that 32.5% attributed this disruption to the limited access to medication.

Following the floods in Iowa, 18.3% of urban households and 16.2% of rural households reported that at least one individual lacked access to a 3-day supply of prescription medications 37.

**Table 4**

**Figure:**
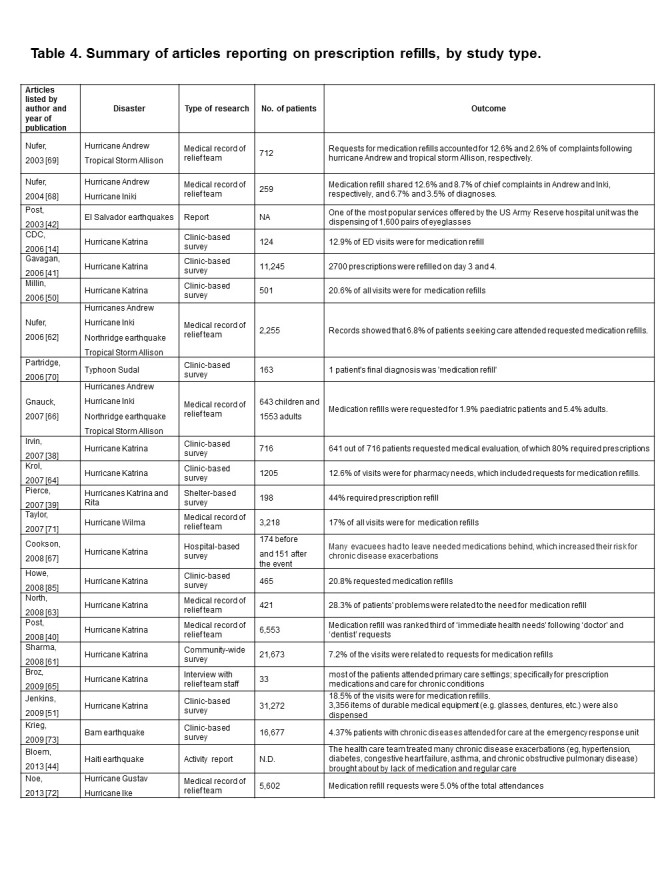

Table 4 is a summary of articles reporting on prescription refills, by study type and lists chronologically the disaster, type of research, number of patients and the relevant outcome from each study.

Twenty-three articles described patients coming to field clinics or emergency hospitals for prescription refills. In each article listed, where available, the frequency of refill request percentage by study is documented. Details of some of the studies and their most relevant findings are summarised briefly below:

- After Hurricanes Katrina and Rita, 88 out of 198 evacuees (44%) at one shelter required a prescription refill 38 ^,^ 39; according to a rapid needs assessment among the Katrina evacuees, medication refill was ranked third in terms of ‘immediate health needs’ following ‘doctor’ and ‘dentist’ 40.
- Gavagan et al. 41 reported on the post Hurricane Katrina evacuation complex in the Houston Astrodome. They found that **c**hronic disease problems or medication refills prompted most adult visits to the clinic and that these refill requests were the fourth most common health and health related issue identified. Of note they showed that obtaining prescriptions for narcotic pain relief medication or refills of implanted narcotic and baclofen pumps proved difficult.
- Medications are not limited to orally-taken pills - after the earthquake in El Salvador in 2001, one of the most popular services offered by the US Army Reserve hospital unit was the dispensing of 1,600 pairs of eyeglasses 42 and the loss and breakage of eyeglasses was also identified as an issue by Sareen and Shoaf 43 following the Northridge earthquake
- Following the Haiti earthquake a health care team survey showed that many chronic disease exacerbations (eg, hypertension, diabetes, congestive heart failure, asthma, and chronic obstructive pulmonary disease) were brought about by lack of medication and regular care 44.

**Table 5**

**Table 5. Examples of the range of medical needs following post disaster evacuation:** 

**Routine medications**	
	E.g. prescription medications for hypertension, diabetes, etc. See Table 4 for references.
**Medical records**	
	List of medications (Clark et al., 2007; Kamoi et al., 2006; Kishimoto and Noda, 2012; Krousel-Wood, 2009; Lach et al., 2005)
	Medication logs (e.g. chemotherapy) (Imamura and Ueno, 2011)
	Laboratory data (e.g. Tuberculosis test results, CD4^+^ T-cell counts for HIV patients) (Fowler et al., 2005)
	Allergy information (Fung and Loke, 2010)
	Style and serial numbers of the devices (e.g. pacemakers) (Lach et al., 2005)
**Devices for specific care**	
	Devices for insulin delivery (e.g. needles, glucose-sensor, cartridge)^,^(Jenkins et al., 2009b; Kamoi et al., 2006; Miller and Arquilla, 2008)
	Nebulizer machines (Jenkins et al., 2009b)
	CPAP machines (Jenkins et al., 2009b)
	Power generator / automobile with inverter for electrical device (Tanaka, 2013)
	Oxygen cylinders/concentrators (Jenkins et al., 2009b)
	Canned nutritional supplements for the tube feedings (Mace et al., 2010)
**Devices for daily life**	
	Glasses (Lach et al., 2005; Sakashita et al., 2013)
	Hearing aids (Lach et al., 2005)
	Canes (Jenkins et al., 2009b)
	Walkers (Jenkins et al., 2009b)
	Wheel chairs (Lach et al., 2005)
	Dentures (Lach et al., 2005; Sareen and Shoaf, 2000)
**Emergency medications**	
	e.g. potassium-binding resin for patients taking dialysis (Foster et al., 2011; Millin et al., 2006)
**Over-the-counter (OTC) medications**	
	Painkillers/medication for fever (Fung and Loke, 2010)
	Anti-histamine agents (Fung and Loke, 2010)
**Others**	
	Personal identifier for those who cannot speak (Andersson et al., 2006)
	Medication opening devices for those with hand disabilities (Mori et al., 2007)

The most challenging part to bringing medication is ensuring a patient carries the full range of medication they need, including medical records, emergency drugs and life-support devices. Examples of the range of medications required following a disaster and evacuation was drawn from the reviewed articles, and is summarised in Table 5.

Medical records are critical in some situations, such as CD4 cell count and HIV RNA levels for HIV patients 24 medication logs for cancer patients 45 and Tb test results 12. Even for other patients, medical records including allergy to medications 24 ^,^ 30 ^,^ 31 ^,^ 46 ^,^ 47 are essential. For patients with specific medical devices such as pacemakers, style and serial numbers of the devices is important 47. It is recommended that individuals keep a list of these essential items , which should be reviewed updated periodically 46 and the list preferably kept it in wallet or purse, which is likely to be brought along during an emergency 48.

Prescriptions specific to emergency situations should also be considered for each patient. For example, dialysis patients need to bring a potassium-exchanging resin, which is essential to reduce the potassium level when the access to dialysis is limited 49 ^,^ 50. Devices for insulin injection (vials, needles or pens with replaceable cartridges of insulin) 31, gluco-metres 4 ^,^ 31 ^,^ 51, fluids and devices for peritoneal dialysis, 20 nebuliser machines, CPAP machines, oxygen cylinders, 51 batteries for aspirators and artificial ventilators 52, suctioning and tube feedings, and canned nutritional supplements for the tube feedings 53 should also be considered as part of the emergency pack for patients to bring. For the families with children who are dependent on technology and electrical devices, it seems more difficult to prepare for all the life-supporting equipment required, such as power generator or car/vans that can be used to generate electricity 54.

Supportive tools for daily life, such as wheel chairs, hearing aids 47, canes, walkers, 51 dentures, glasses 43 ^,^ 47, extra batteries for wheelchairs and other assistive devices, and incontinence briefs for the elderly 47 are often lost at the time of evacuation. For those who cannot speak, bringing a personal identifier is also critical 55. Over-the-counter medicines, such as medication for fever or pain, anti-histamine for allergy, denture adhesive, and sanitary products are also important when access to pharmacies are disrupted 56. For those with hand disabilities, openers for the medications are also an essential item 57.

## Discussion

This systematic review revealed that a considerable number of patients lose their medication during evacuation. As a result, medication refill is an immediate health need, making the prescription of medications for pre-existing conditions an increasing burden of medical relief activities at a time when acute needs are also over-whelming. At the current time, preparedness with respect to medications for disasters is not fully appreciated nor given much attention by those requiring daily and constant medication; meaning that a large number of patients facing extreme events could have avoided prescription interruption had they not lost their medication and or medical devices. Until individuals, with the assistance of their healthcare providers, undertake preparative actions, those organising relief activities need to be prepared to cope with emerging treatment alongside the management of chronic illnesses, including medication refills and devices.

The discussion is presented by a) the impact of medication loss and interruption of care, b) the impact of prescription refill post disaster and c) the value of effective preparation actions.

**a) The impact of medication loss and interruption of care**

Studies from developed countries show that the impact of medication loss and interruption of care can be a significant issue. From the results above, it is possible to demonstrate that surveys targeting paediatric patients in New Orleans after Hurricane Katrina revealed that 33.9% of evacuated children with pre-existing chronic conditions ran out of medication; 26 as a result, 58.4% experienced at least one disruption to care. In a questionnaire-based survey of geriatric patients visiting a hospital in Florida one year after Hurricane Wilma, 3.4%-6.7% reported that they had missed medication within two weeks of the event 34. Some studies found that although some people brought their medication with them upon evacuation, they had only brought enough supply for a limited period 58. After Hurricane Ivan in 2004, 10% of households in most affected counties had problem obtaining medication one week after the storm, thus bringing sufficient supply may have be difficult 32. After the Great East Japan Earthquake and the following tsunamis in 2011, many evacuees had no time to gather their belongings. Some of them were treated with unique medication, such as immuno-suppressants, which were not obtainable at the disaster area. As a result, these patients had to stop their medication for weeks knowing that their medical conditions would deteriorate 59.

The problem regarding medication loss is not limited to developed countries. Among the internally-displaced population from the 2005 Pakistan earthquake, 85% of the households in small unofficial camps had no available drugs to manage their chronic medical conditions. After the Haiti earthquake in 2010, a relief team reported treating many women for chronic disease exacerbations brought about by lack of medication and/or regular care 44.

Some articles reported medication by specific chronic condition, which showed that the level of preparation may vary by conditions. People with mental illness are also at high risk of medication interruption 60 ^,^ 1.

Examples of loss of medication lists and medical devices were identified as medical and life-support devices are as important as technological tools like haemodialysis and oxygen. A questionnaire conducted on the evacuees two weeks after Hurricane Katrina revealed that medical services were only the fourth most commonly reported medical need. The most common was dental care (57% of the respondents), followed by eyeglasses (34%) and dentures (28%). Other devices needed were hearing aids, canes, wheel-chairs, and walkers 16.

**b) The impact of prescription refill post-disaster**

The high prevalence of the need of prescriptions becomes a burden on medical teams at the disaster area which was identified in Table 4. For example, an analysis of survey data after Hurricane Katrina revealed 7.2% of the patients visiting emergency treatment facilities within 2 months of the hurricane were attending for medication refill 61.

Looking at the timeline of health needs following a disaster, the proportion of medication refills does not appear to change between the early and late stage of the relief activities. For example, according to a retrospective review on the patients seen by a Disaster Medical Assistance Team during four extreme events in New Mexico, US, the proportion of the patients visiting for medication refill was 6.0% within 7 days from the events and 7.6% after 7 days 62. Even for specialty care, medication refill often shares a significant part of relief activities. After Katrina, of 421 patients who were seen by mental health professionals, 119 (28.3%) were attending for medication refill 63.

Despite medication refills being a common need among the disasters studied, the proportions of patients coming to health facilities for medication refill vary within and between events. For example 20.9% in a Louisiana clinic site two weeks after the event, 50, within 2 months 20.8% in a temporary clinic in New Orleans 24, 12.6% in the mobile medical units within 3 weeks, 64 and 48% in medical units in Chicago between 1-4 weeks after the event. 65

Additionally, a comparison between disasters suggested the burden differs depending on the scale and types of the disasters 66 For example, Cookson et al. 67 reported that non-significant increases were seen with medication refill request after the Katrina. In other cases, the proportion requiring medication refill was reported to be 3.5-3.6% after the Hurricane Iniki in 1992 66 ^,^ 68, 6.7%-10.0% after the Hurricane Andrew in 1992 66 ^,^ 68, 1.3-3.7% after Tropical Storm Allison in 2001, 66 ^,^ 69 0.6% after Typhoon Sudal in Micronesia in 2004 70, 7% after the Hurricane Wilma in 2005, 71 and 4.8-7.1% after Hurricanes Gustav and Ike in 2008 72. Another example from the Bam Earthquake in Iran in 2003 showed that the management of ‘chronic disease under treatment’ when measured required only 4.37% of the tasks of the emergency response unit. 73.

**c) The value of effective preparation actions**

Although the main scope of the review was to identify patients’ reactions relating to bringing their medications during a disaster, several implications for effective preparedness were identified.

Twelve articles described possible effective preparation actions for patients. Having a personal stockpile is recommended in many articles, though the recommended personal stockpile ranges from 3-4 days 20 ^,^ 74 to 1 month 75. In a survey study conducted in California, US, the proportion of those who had a 2-week supply of medication ranged from 60.1% among non-veteran women to 81.9% among veteran men 76. It was customary in India for pregnant women (81.1% reported) to have individual stockpile of their routine medications 77.

However, just keeping extra doses of medication stockpiled is likely to have limited impact. In a hospital-based survey study targeting evacuated outpatients from the Japanese flash flood in 2005, keeping a personal stockpile did not increase the likelihood of bringing medications to the evacuated sites 19. On the other hand, those who had prepared an emergency pack were 5.7 times more likely to bring medications to the evacuated sites 19. Therefore, the researchers recommended that the stockpile is packed in a bag for easy access. Even so, the compliance for making an emergency pack seems to be low. For example, 63% of the haemodialysis patients in California had a 2-week supply of medicines but only 31% stored the items in an emergency pack. 49 In a study targeting patients with rheumatoid arthritis in Japan, 46% reported they had a personal stockpile of their medications but only 25% had packed an emergency bag 78. Other researchers reported that although 82.8% of households with children had stocked common medication for fever or pain for 3days, only 60.6% kept a first aid box and only 14.6% thought they would take their medications during evacuation 56.

Carrying medications at all time 58 ^,^ 78 ^,^ 79 or keeping extra medication in multiple places, such as schools and offices 80, are described as the most robust and effective emergency plan for patients. However, low compliance with this action has been reported. Among the rheumatoid arthritis patients in Japan, only 53% of those who had a personal stockpile carried their medication all the time 78. Among HIV patients, 33% of patients did not have individual health cards at the time of interview, potentially making the provision of therapy to these patients difficult 19 ^,^ 79.

Although having a personal stockpile is recommended by the Centres for Disease Control and Prevention (CDC) and the American Red Cross 81, our review implies that just having a personal stockpile might not be effective 78. Many onsite workers recommend patients should have an emergency bag. For example, a station manager at The Hampshire Fire & Rescue Service in the United Kingdom (UK) remembered: ‘after fires, many people had to go back to their home to get medications and medical devices they need.’ He said that if communities can be educated in advance they will be able to prepare an emergency ‘grab bag’ containing vital personal items such as prescription medicines and medical devices and glasses/contact lenses, and suggests that this increases the likelihood of bring medicines/devices during a disaster 82.

To achieve patients’ preparedness, healthcare professionals play an essential role in establishing effective emergency planning for patients.

- First of all, they should provide patients with medications and other resources sufficient for disaster preparedness as well as up-to-date medical records. 30 A successful case study can be taken from the time of the election violence in Kenya 79, during which the dispensing of greater quantities of prescriptions were protective against treatment interruption among HIV patients. 79
- Secondly, health professionals have a responsibility to educate patients about the potential health impacts of medication interruption, and can emphasise that bringing medication and medical records may be the only way to enable them to continue normal care in an emergency.
- Thirdly, they must help patients to design an individualised and practical emergency plan that takes in to account patient-specific barriers such as forgetfulness, 58 side effects 8, and allergy 83. In addition, they can help train patients in practices of safe storing medicines and packing necessary medical devices^75^: the medication and medical devices in the emergency pack should be effectively protected from contamination by toxins from flood waters^84^ or mechanical damage by an earthquake 43.
- Fourthly, in disasters for which there is some advance warning, such as hurricanes, health professionals may make contact with patients when a disaster alert has been made, reminding them to bring their medication and medical records when they evacuate 58 ^,^ 74.
- Finally, and most importantly, front-line public health workers and the members of rescue teams should have adequate medication for their own medical conditions to sustain them for the duration of their rescue efforts during a disaster 56 ^,^ 85.

Other stakeholders, such as policy makers and researchers, should also coordinate around patients’ emergency planning (Figure 2). Recently, an increasing effort has been made by national and local governments in several countries to encourage patients to prepare an emergency bag. For example, in the UK, the National Health Service provides discharged patients with a carry bag (‘green bag’) for their medications to encourage patients to bring their medication in emergency situations 82, which is applicable to the time of evacuation. In the US, the CDC and American Red Cross raised ‘gather emergency supplies’ as the first step for emergency preparedness 85 ^,^ 86.

**Recommendations on how community members can prevent medication losses:**
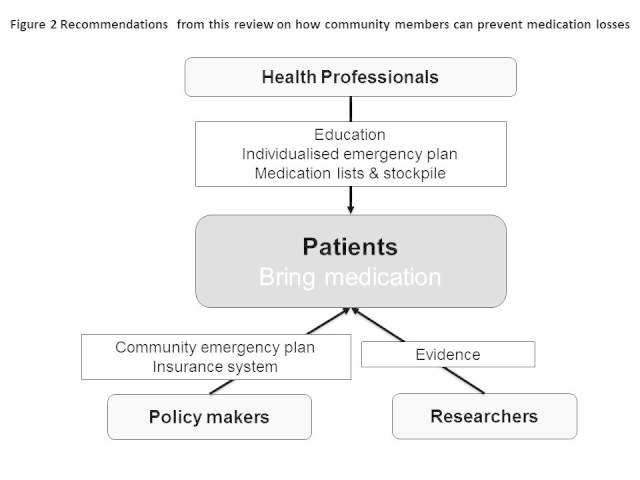

Even so, evidence is still weak with regard to preparedness actions by patients and the efficacy of intervention to encourage preparation. Researchers should be actively involved in disaster plans to leverage the preparedness among patients. For example, health impacts caused by loss of medication should be assessed using feasible and standardised methods to enable targeted aid following a disaster. Baseline data should also be measured and made available to appropriate agencies, including the burden of chronic diseases in each community 87 ^,^ 88, health care disparities 89, vulnerable populations for whom preparedness is a challenge 90, preparedness of general or specific groups of people 34 ^,^ 37 as well as factors that affect emergency preparedness 91. Intensive research following disasters is also critical, and should include rapid health needs assessment among the evacuees, 37 ^,^ 61 health impact assessment including medication adherence 92, and evaluation of the efficacy of preparedness actions 90 to inform future planning and preparation.

The key findings and recommendations are summarised in Box 1.

**Figure:**
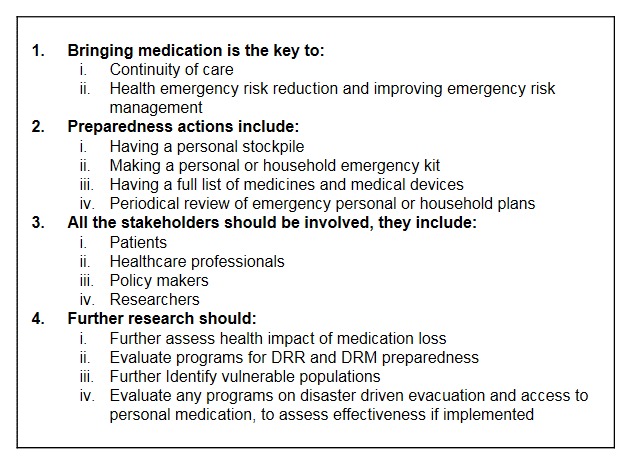
**Box 1. Summary of key findings** DRR – disaster risk reduction; DRM – disaster risk management

**Limitations**

The most significant limitation of this study is lack of comparative data. There is no standardised way of measuring the impact of bringing medication at evacuation. Most frequently, survey is conducted on convenience samples, which makes it hard to generalise beyond that particular population. After a disaster, obtaining quality data is challenging due to the flow of evacuees and temporary nature of their status, lack of personnel, and ethical concerns accompanying the conduct of research on suffering people. Simple, unobtrusive and feasible approaches of monitoring preparedness and health outcomes should be carefully designed and established before disasters occur, especially in those regions subject to frequent disasters.

Another limitation is publication bias; most of the relevant articles were from the US, and a large proportion specifically focused on hurricane Katrina. Whether the issue of medication loss is less a problem in developing countries or simply less frequently studied and/or published is not clear. There is a clear need for evidence from all over the world, and from the most marginalised, thus rarely reported, populations.

This research does not focus on longer term crises, such as drought or political and economic failures, in which restoration of healthcare provision may take many months 5. In such disasters, other issues may predominate, and preparing and bringing a stockpile of medication/medical devices may not be the best solution.

**Conclusion**

To achieve patients’ preparedness, healthcare professionals play an essential role in establishing effective emergency planning for patients should provide patients with medications and other resources sufficient for disaster preparedness as well as up-to-date medical records. Health professionals have a responsibility to educate patients about the potential health impacts of medication interruption, emphasising that bringing medication and medical records may be the only way to enable them to continue normal care in an emergency. They must help patients to design an individualised and practical emergency plan that takes in to account patient-specific barriers such as forgetfulness. In disasters for which there is some advance warning, such as hurricanes, health professionals may make contact with patients reminding them to bring their medication and medical records when they evacuate. Front-line public health workers and the members of rescue teams should have adequate medication for their own medical conditions to sustain them for the duration of their rescue efforts during a disaster.

People may survive the initial disaster but if they are not educated or appropriately prepared in particular when medication is involved they may not survive the aftermath.

## Appendix 1

### PRISMA Checklist

Download PDF
